# Learning From International Comparators of National Medical Imaging Initiatives for AI Development: Multiphase Qualitative Study

**DOI:** 10.2196/51168

**Published:** 2024-01-04

**Authors:** Kassandra Karpathakis, Emma Pencheon, Dominic Cushnan

**Affiliations:** 1 Decimal.health Boston, MA United States; 2 Foreign, Commonwealth and Development Office UK Government London United Kingdom; 3 NHS England London United Kingdom

**Keywords:** digital health, mobile health, mHealth, medical imaging, artificial intelligence, health policy

## Abstract

**Background:**

The COVID-19 pandemic drove investment and research into medical imaging platforms to provide data to create artificial intelligence (AI) algorithms for the management of patients with COVID-19. Building on the success of England’s National COVID-19 Chest Imaging Database, the national digital policy body (NHSX) sought to create a generalized national medical imaging platform for the development, validation, and deployment of algorithms.

**Objective:**

This study aims to understand international use cases of medical imaging platforms for the development and implementation of algorithms to inform the creation of England’s national imaging platform.

**Methods:**

The National Health Service (NHS) AI Lab Policy and Strategy Team adopted a multiphased approach: (1) identification and prioritization of national AI imaging platforms; (2) Political, Economic, Social, Technological, Legal, and Environmental (PESTLE) factor analysis deep dive into national AI imaging platforms; (3) semistructured interviews with key stakeholders; (4) workshop on emerging themes and insights with the internal NHSX team; and (5) formulation of policy recommendations.

**Results:**

International use cases of national AI imaging platforms (n=7) were prioritized for PESTLE factor analysis. Stakeholders (n=13) from the international use cases were interviewed. Themes (n=8) from the semistructured interviews, including interview quotes, were analyzed with workshop participants (n=5). The outputs of the deep dives, interviews, and workshop were synthesized thematically into 8 categories with 17 subcategories. On the basis of the insights from the international use cases, policy recommendations (n=12) were developed to support the NHS AI Lab in the design and development of the English national medical imaging platform.

**Conclusions:**

The creation of AI algorithms supporting technology and infrastructure such as platforms often occurs in isolation within countries, let alone between countries. This novel policy research project sought to bridge the gap by learning from the challenges, successes, and experience of England’s international counterparts. Policy recommendations based on international learnings focused on the demonstrable benefits of the platform to secure sustainable funding, validation of algorithms and infrastructure to support in situ deployment, and creating wraparound tools for nontechnical participants such as clinicians to engage with algorithm creation. As health care organizations increasingly adopt technological solutions, policy makers have a responsibility to ensure that initiatives are informed by learnings from both national and international initiatives as well as disseminating the outcomes of their work.

## Introduction

### Background

Medical imaging has been identified by many governments as an especially promising application for artificial intelligence (AI) in clinical practice with the potential to enhance disease screening, improve care outcomes, and reduce costs [1-5]. Optimizing AI capabilities requires aggregating and streamlining access to medical imaging data for machine learning (ML) model training and validation and contextualized mechanisms for deployment in clinical workflows.

During England's National Health Service (NHS) response to the COVID-19 pandemic, the digital health agency (NHSX) created the National COVID-19 Chest Imaging Database (NCCID). The NCCID is a “centralized UK database containing chest X-rays (CXR), Computer Tomography (CT) and Magnetic Resonance Images (MRI) from hospital patients” with COVID-19 [6,7]. It was established to develop, validate, and deploy AI and ML models for supporting the management of patients with severe COVID-19. The creation of the NCCID highlighted the merits and challenges of a centralized approach for collating national imaging data [7].

The NCCID led to a proposal for a generalized national imaging platform for the development, validation, and deployment of AI and ML models in medical imaging. This platform was envisaged to have three technical functions:

1. A data pipeline to facilitate the collection of data nationally
2. A trusted research environment (TRE) to provide access to national data to build and validate new AI and ML products
3. A deployment platform to act as an “app store” for the most up-to-date AI and ML models for users in health care facilities

To support the safe, ethical, and effective creation and deployment of a national imaging platform, the NHS AI Lab developed complementary policy and regulatory initiatives, including a cross-regulatory service to guide developers through the regulation of their AI products [8], understanding of public attitudes toward sharing health data for AI development, and an Algorithmic Impact Assessment tool to identify potential societal impacts of AI products [9].

Beyond understanding the policy and infrastructural requirements, it is important to assess the strengths and weaknesses of such a national approach to produce AI and ML models for imaging that can be deployed in clinical workflows. To make such an assessment, the NHS AI Lab analyzed international efforts to build similar medical imaging platforms in both private and public organizations, some of which were associated with national efforts to diagnose and manage patients with COVID-19. The NHS AI Lab used the outputs of the research to understand the approaches taken and lessons learned and inform the design of England’s national imaging platform.

### Objectives

We sought to identify and understand international use cases of and proposals for medical imaging platforms to streamline the innovation-to-deployment journey for health AI models in imaging. We aimed to understand how imaging for AI efforts were structured, identify the constituent parts of the initiatives (eg, technical aspects, users and marketplace, and commercialization), and understand the implications of government policy and regulation. We used this analysis of international use cases to formulate policy recommendations for England’s nascent national AI imaging platform.

## Methods

### Overview

This research was conducted by NHSX, the former digital health agency and technology policy arm of NHS England. NHSX was merged into the NHS England transformation directorate in 2022. The Strategy and Policy Team at the NHS AI Lab, which was embedded inside NHSX, led and completed the study. This project was conducted between September 2020 and March 2021.

### Phase 1: Identification and Prioritization of National AI Imaging Platforms

We conducted a preliminary scan to identify efforts to create national AI imaging platforms in other countries that the NHS AI Lab could analyze in depth.

As the United Kingdom was poised to lead the G7 in 2021, we started with fellow G7 countries: Canada, France, Germany, Italy, Japan, and the United States of America. We then scanned non-G7 countries known within digital health policy circles for their digital health approaches or that had previously responded to an NHSX survey on the use of AI by Global Digital Health Partnership (GDHP) member countries [10]: Australia, Brazil, China, Estonia, Hong Kong, India, Republic of Korea, Rwanda, Singapore, Sweden, Uganda, and Uruguay. Finally, we scanned initiatives in multilateral collaborations (World Health Organization, International Telecommunication Union, and the GDHP) and major private organizations (eg, GE Healthcare and Google).

National AI imaging initiatives were identified by 2 researchers (Abhishek Mishra and EP) through (1) a targeted Google search for each country using *[country]* and the keywords *AI medical imaging platform*, *medical imaging data*, *medical AI platform*, *AI radiology*, or *COVID-19 medical image AI*; (2) a targeted Google search for multilateral collaborations and major private organizations using *[name of organization]* and the keywords *AI medical imaging platform*, *medical imaging data*, *medical AI platform*, *AI radiology*, or *COVID-19 medical image AI*; and (3) a general search on Google, Google Scholar, Twitter, and One HealthTech using the keywords *medical imaging AI platform*, *medical imaging platform*, *national medical imaging AI platform*, or *medical imaging AI marketplace*. For each search, the first 5 pages of the results were scanned owing to time and resource limitations.

We scored each initiative in comparison with the United Kingdom’s context to prioritize some for the deeper dive in phase 2. Each of the following criteria (n=4) was scored from similar (score=3) to not similar (score=1); initiatives with the highest total score were deemed most similar to that of the United Kingdom:

1. Similarity of the medical imaging platform to the United Kingdom’s proposed initiative: medical imaging data only versus additional health data, TRE built on top of data to allow for model development, data consolidated in a centralized location or alternative approaches such as federated learning, and parallel building of deployment platform.
2. Size of market: using the country population as a proxy — ≥50 million, 10 to 50 million, and 0 to 10 million.
3. Future trade importance to the United Kingdom: priority markets identified by the NHS Director of AI based on track record of digital health initiatives (note that, at the time of the study, the United Kingdom was the Chair of the G7, and there was strong political interest in the potential for health AI to bolster the United Kingdom’s trade agenda).
4. Regulatory and ecosystem similarity to that of the United Kingdom based on the following: provincial versus national digital health organization, single-payer versus multipayer system, and regulatory approach to AI.

### Phase 2: Deep Dive Into National AI Imaging Platforms

For the prioritized initiatives, we conducted a deep dive using the Political, Economic, Social, Technological, Legal, and Environmental (PESTLE) factors framework. PESTLE is a common tool used in policy analysis to gain an overview of an industry [11].

The aims of the deep dive were to (1) identify reliable and robust information to inform the understanding of the international use case; (2) identify hypotheses, gaps, and insights on the AI imaging initiatives for validation during stakeholder interviews; and (3) inform the creation of a deductive framework for the analysis of semistructured interviews. We also identified stakeholders leading AI initiatives to approach for the semistructured interviews in phase 3.

### Phase 3: Semistructured Interviews

Semistructured interviews were conducted to understand each prioritized initiative (eg, data used and intended users); its social and political context (eg, regulatory landscape, stakeholders, and public trust), data handling (eg, data and privacy laws), funding sources, and commercialization; and the lessons learned during its development. The discussion guide (Multimedia Appendix 1) was tailored to each country’s unique imaging platform, including the validation of any gaps or insights identified in phase 2.

The interviews were conducted by one principal researcher (KK) with one supporting researcher (EP). Informed consent was obtained from interview participants, and they approved the selected quotes for publication. The interviews lasted up to 1 hour and were audio recorded, and detailed notes were taken. Transcription and translation services were provided by an independent agency. Only one country (Singapore) required the use of translation services to conduct the interview. All other interviews were conducted in English. Both the detailed notes and transcripts from the interviews were analyzed.

The interviews were analyzed using a deductive framework with codes identified from the desk research deep dives (Multimedia Appendix 2). In total, 2 researchers (KK and EP) analyzed each interview independently and compared their coding. Intercoder reliability (ICR) was calculated to assess the reliability of the coding protocol and thematic analysis. ICR was calculated by comparing the level of agreement and disagreement across the coding for 5 pages per transcript [12].

### Phase 4: Workshop With NHS AI Lab National Imaging Platform Team

A workshop was conducted with the NHS AI Lab national AI imaging platform team members who were conducting the discovery phase [13]. The workshop aims were to (1) establish top areas of interest from the perspective of the discovery team, (2) explore why these areas are important to the team, and (3) stimulate the discovery teams’ interest in applying the lessons learned from other countries.

The workshop was facilitated by one principal researcher (KK) with one supporting researcher (EP). The workshop lasted 90 minutes, and audio recordings and detailed notes were taken. Participants (n=5) used the web-based *Padlet* and *Jamboard* (Google) post-it and “like” functionalities. If required, the researchers noted the participants’ points on their behalf. The workshop audio was transcribed and analyzed.

An overview of the initiatives (n=6) from phase 2 and phase 3 was provided to the attendees using *Jamboard*. The countries were treated as individual case studies rather than grouped together because of the large degree of heterogeneity between the countries.

A total of 8 themes from the deductive framework were used to guide the workshop: purpose; users; organizational; commercialization; data; incentives; building trust; and law, policy, and regulation. Quotes from the semistructured interviews with stakeholders (phase 3) from each initiative were mapped to the 8 themes for discussion at the workshop.

The nominal group technique was used to identify priority quotes and insights [14]. Participants were asked to vote on the quotes that resonated or were of interest to them using *Padlet*’s “like” functionality. Each participant had 6 votes per initiative. Voting indicated the discovery team’s priorities and fueled discussions.

The outputs of the deep dives, interviews, and workshop were synthesized thematically into 8 categories with 17 subcategories. The analysis was inspired by a user-centered design insight format [15], which states the context and background, explains the learning, explains the root cause (the why), and explains the motivation behind why the learning has occurred and the ramifications for the NHS AI Lab’s proposed national medical AI imaging initiative.

### Phase 5: Formulating Recommendations

The researchers (KK and EP) jointly synthesized all the data gathered from phase 3 to phase 4 to formulate recommendations for the NHS AI Lab national AI imaging initiative. This involved drawing out themes based on the original thematic framework, identifying learnings pertinent to the United Kingdom, and framing the resulting insights into actionable recommendations.

Final recommendations were presented to the Head of AI Imaging and Director of AI at the NHS AI Lab for consideration. The Head of AI Imaging and the national AI imaging discovery team selected the recommendations that were relevant and actionable for the discovery and future phases of the project. The research team was not privy to this selection.

### Ethical Considerations

Internal and external stakeholders were consulted during this policy research and development. Informed consent was obtained from interview and workshop participants. Per NHSX’s standard practice, independent ethical review was not required for this research informing policy as it poses negligible risk.

## Results

### Phase 1: Identified National AI Medical Imaging Platforms

Numerous initiatives (n=34) were identified from preliminary scanning. Most initiatives were country based (21/34, 62%), and the remainder were from major private organizations (10/34, 29%) or multinational organizations (3/34, 9%). Some of the initiatives (7/34, 21%) were prioritized for a deep dive: (1) Digital Health and Discovery Platform (DHDP; Canada), (2) national medical image database (China), (3) Hospital Authority Data Collaboration Laboratory (HADCL; Hong Kong), (4) Research Center for Medical Big Data (Japan), (5) AI Medical Imaging Platform (Singapore), (6) Analytic Imaging Diagnostics Arena (AIDA; Sweden), and (7) Medical Imaging and Data Resource Center (MIDRC; United States).

### Phases 2 and 3: Overview of Prioritized National AI Imaging Platforms

In the following sections, we provide a brief overview of each initiative. Multimedia Appendix 3 [16-44] provides a detailed overview of each country’s initiative complemented with findings from the PESTLE analysis and semistructured interviews.

#### Canada: DHDP

This pan-Canadian initiative was set up to create a nationwide framework to digitally enable research that advances next-generation precision medicine technologies with an emphasis on cancer and improving health outcomes for patients. The DHDP comprises >90 consortium partners spanning academia and the private sector. The initiative focused on numerous types of medical data rather than solely on medical imaging [45] and undertook novel research in federated learning technologies that reflected Canada’s stringent attitudes toward data privacy and sharing.

#### China: National Medical Image Database

In September 2020, plans were announced for the creation of a standardized national medical image database. The Chinese national medical image database was approved by the National Health Commission [19] to enable hospitals to share patient information and medical images and support the training and development of AI technology for health care. At the time of the study, it was unclear what technology stack the Chinese national imaging database would use and how the initiative would overcome issues of data digitization, cybersecurity, and commercialization.

#### Hong Kong: HADCL

The HADCL was established to support the formulation of health care policies, facilitate biotechnological research, and improve clinical and health care services. The HADCL is the flexible and interactive data-sharing channel of Hong Kong’s Hospital Authority, with a growing focus on the development of AI and ML algorithms. It is a full-service offering encouraging researchers to partake in collaborative health data projects in a controlled environment using the Hospital Authority’s extensive, longitudinal data [46,47].

#### Japan: Research Center for Medical Big Data

Japan’s Research Center for Medical Big Data is a platform for AI technology research and development, including a cloud-based platform for hosting medical imaging big data and analyzing medical images. As of 2019, the platform contained >10 million medical images, with participation from at least 60 hospitals. In line with policy at the time of the study, the platform’s primary user base was academia, and projects were for research purposes only.

#### Singapore: AI-Enabled Medical Imaging Platform

In October 2020, the Integrated Health Information System health laboratory issued a call for collaboration between partners to cocreate an “AI-enabled Medical Imaging Platform” aimed at operationalizing and exploring AI models and applications for medical imaging. The platform will be open and vendor neutral, thereby enabling the deployment of AI models and products from different sources to assist with clinicians’ work.

#### Sweden: AIDA

AIDA is a dedicated initiative for research and innovation in AI and medical image analysis in Sweden. The initiative brings together academia, health care, and industry to translate innovation into AI-based decision support solutions for imaging diagnostics. The previous mandated creation of national registries containing >5 TB of health data provided the foundation for the AIDA initiative.

#### United States: MIDRC

The MIDRC is a multi-institutional initiative established in response to the COVID-19 pandemic. The aim was to foster ML innovation through the sharing of imaging and associated clinical data regarding COVID-19 [48]. At the time of the study, agreements for sharing relevant medical imaging data were in the process of being signed with several sites, but no data were being hosted on the platform.

### Phases 3 and 4: Derived Themes and Insights

Stakeholders (n=16) representing 7 initiatives were approached for interviews. Stakeholders (n=13) from 6 initiatives accepted the interview invitations (13/16, 81% acceptance rate). The stakeholders from participating countries were 38% (5/13) from Canada, 8% (1/13) from Hong Kong, 23% (3/13) from Japan, 8% (1/13) from Singapore, 8% (1/13) from Sweden, and 15% (2/13) from the United States. Stakeholders from China (3/16, 19%) did not respond to the request for an interview.

For the interview coding, the ICR between the researchers (KK and EP) was calculated to be 0.41, indicating moderate reliability [12,49]. The outputs of the deep dives, interviews, and workshop were synthesized thematically into categories (n=8) with subcategories (n=17).

Multimedia Appendix 4 presents the categories, subcategories, and corresponding thematic synthesis within each of the other countries’ initiative including key insights, quotes, and learnings.

### Phase 5: Recommendations

#### Overview

We provided 12 recommendations for the NHS AI Lab’s proposed national AI imaging platform. Each recommendation is grounded in the themes and insights from phase 2 to phase 4 (see Multimedia Appendix 4). The corresponding themes for each recommendation are also provided.

#### Narrative

Recommendation 1: The NHS AI Lab develop a purposeful narrative of why and how a national medical imaging initiative is necessary, outlining what health needs it will meet and supporting this with demonstration of its benefit and potential

Developing a strong value proposition should be married with demonstrable benefit. The narrative should be cross-cutting, speaking not only to purpose but also to trust and incentives, with transparency regarding the drivers of the initiative. Previous work by the NHS AI Lab on behalf of the GDHP has also argued that countries should take a “needs based” approach to AI-driven technology development to create both maximal benefit on health outcomes and foster buy-in and support from stakeholders and the public [47,50].

A purposeful narrative for the NHS AI Lab’s national medical imaging initiative will support interdisciplinary collaboration and ensure long-term political, financial, and social support for the initiative based on a clear understanding of its importance and utility to the health system. An important aspect of this narrative is to reference the value of the initiative as a social or public good that creates public value [51].

The corresponding themes for this recommendation are (A) demonstrable benefit of the initiative, (B) health system needs as the primary driver, (C) community and shared purpose, and (O) transparency and communication. transparency and communication.

Recommendation 2: The NHS AI Lab moves away from the language of “platform” to talking about the national medical imaging initiative as an “initiative” and community space for growing the United Kingdom’s understanding and ability to use AI in medical imaging

The United Kingdom’s national medical imaging “initiative” should be carefully framed, using language that reflects what is offered and conveys mindset and purpose. The connotations of “national” in the initiative name given the involvement (or lack thereof) of the Devolved Administrations (DAs) should be considered. In addition, the NHS AI Lab should develop an approach for involving the DAs.

The corresponding themes for this recommendation are (C) community and shared purpose and (D) embracing and enabling the central role of health care professionals.

#### Users and Service Offering

Recommendation 3: The NHS AI Lab develops wraparound services to maximize engagement and capitalize on the expertise of varied users; by removing the need to technically upskill in AI development while also providing opportunities for users to do so if they wish, the initiative can broaden participation and avoid disincentivizing users with different and valuable areas of specialty

The NHS AI Lab should invest in wraparound services, specifically offering tools and professional technical skills that are tailored to fill a gap that users, such as health care professionals, have when it comes to developing AI. It appears from international comparators that the main draw and success has not been the platform itself but the supportive services to enable users to engage, collaborate, and develop AI-driven technologies regardless of their technical expertise. Examples include but are not limited to clinical fellowships on health data, networking or pairing clinicians with data scientists, training courses on what is AI and how to develop models, and low-code and no-code AI model development tools. The NHS AI Lab should explore opportunities to build these wraparound services from existing programs in the digital health ecosystem.

The corresponding themes for this recommendation are (D) embracing and enabling the central role of health care professionals, (E) recognizing that users are not discrete groups, and (F) importance of wraparound services.

Recommendation 4: The NHS AI Lab continues to embrace interdisciplinary work while designing, developing, and implementing the national medical imaging initiative; the inherent tensions and perspectives between disciplines are needed to deliver on health system needs

Interdisciplinary work is central to harnessing the breadth of expertise required to build and sustain an initiative that truly addresses health system needs. This means embracing the central role of health care professionals and ensuring the participation of people who have a system view of health and social care, as well as those with frontline experience who will be the ultimate end users of any AI products developed on the platform. Prioritizing user-centered design and health care professionals’ experience means that technical expertise must take an important facilitative and instructive role to both guide and learn from health care professionals about how to leverage AI-driven technologies in the health system. By facilitating interdisciplinary work, radiologists’ expertise can be applied to shore up the quality and appropriateness of the imaging data used. We recommend that active steps be taken to foster collaborative working relationships across disciplines drawing on lessons for interdisciplinary collaboration outlined by Blandford et al [52] and on the examples of activities run in Sweden and Japan.

The corresponding themes for this recommendation are (B) health system needs as the primary driver for AI development, (D) embracing and enabling the central role of health care professionals, and (E) recognizing that users are not discrete groups.

#### Sustainability and Future-Proofing

Recommendation 5: The NHS AI Lab consider the financial sustainability of the national medical imaging initiative from the outset and how this maps to the proposed commercial model

All the international comparators who did not have a clear commercial model raised concerns about financial sustainability. It is worth bearing in mind that demonstrable benefit does not guarantee enduring government support with respect to funding. We recommend that the NHS AI Lab national medical imaging initiative considers how the work will be sustained beyond current funding and ensures that options for commercialization are not excluded by virtue of how the initiative is designed (ie, data-sharing arrangements that preclude commercialization). For the NHS AI Lab’s national medical imaging initiative to have longevity, it is important to keep as many commercial options on the table as possible, including generating revenue from certain aspects of the initiative and exploring public-private partnerships. This could include providing data subsets to fulfill specific needs, such as validation, that can be commercialized as a distinct offering.

The corresponding themes for this recommendation are (I) ensure financial sustainability, (J) differing or absent commercial models, and (L) subsetting data offerings.

Recommendation 6: The NHS AI Lab continues to explore different commercial models for the national medical imaging initiative with a focus on how it might commercialize aspects of the initiative rather than taking an all-or-none approach

Commercialization is likely necessary to ensure the financial sustainability of the initiative. Commercial models were an afterthought for many international comparators, who conveyed the sense that commercialization was viewed as being in opposition to the public good. We recommend thinking about commercial options early on, not only from a practical perspective of building the initiative with this in mind but also to construct a narrative that can interweave commercialization and private sector involvement with the public good. The NHS AI Lab should continue working with internal teams (ie, the NHSX Centre for Improving Data Collaboration) to ensure that the NHS gains fair value for the public from commercial arrangements.

The corresponding themes for this recommendation are (I) ensure financial sustainability, (J) differing or absent commercial models, and (N) a focus on public and social good.

Recommendation 7: The NHS AI Lab explore and potentially adopt some of the future-proofing mechanisms used by international comparators

Sweden and the United States exemplified ways to future-proof data-sharing mechanisms, including specific clauses in data-sharing agreements that granted them the power to revoke data access or extend it to future offerings. This is important for safeguarding against issues further down the road and streamlining the process of setting up data-sharing agreements. Sweden was cognizant that currently, anonymized data might become reidentifiable with advances in data analysis and wanted to mitigate this risk from the outset through the ability to revoke access at any time. We also recommend that, if and where possible, the initiative infrastructure is future-proofed and reusable so that it will be fit for purpose in years to come and offer benefits to other similar initiatives.

The corresponding themes for this recommendation are (M) future-proofing mechanisms for data sharing and (N) a focus on public and social good.

Recommendation 8: The NHS AI Lab balances the need to deliver at pace with the up-front investment of time and effort required to ensure that the resulting initiative is sustainable and future-proofed

A variety of pressures to deliver at pace were identified by international colleagues, which at times nudged countries toward “kicking the can down the road” when it came to thorny challenges such as commercialization. Although a certain level of pace is necessary to demonstrate benefit and garner support, this should be tempered to ensure an up-front investment of time and effort that delivers sustainable returns.

The corresponding themes for this recommendation are (A) demonstrable benefit of the national medical imaging initiative and (G) tempering the pace of development.

Recommendation 9: The NHS AI Lab consider under what conditions it would be acceptable and feasible to move beyond human-in-the-loop approaches in the national medical imaging initiative’s resultant AI-driven technologies

All countries maintained the need for a human to be “in the loop” to ensure the safety, accountability, and acceptability of AI development and products. *Human-in-the-loop* refers to models that require human interaction, whereby human oversight can intervene and determine the outcome of a process or event. However, there is an undertone that moving beyond human-in-the-loop approaches is the future state of AI-driven technology in health and care (in some conditions, not yet defined). We recommend that the NHS AI Lab start considering not only the safety and accountability of systems without humans and when this would be deemed appropriate but also the public perception of not having unique or individualized care.

The corresponding themes for this recommendation are (K) common and continuing data challenges, (O) transparency and communication, and (P) keeping humans in the loop.

Recommendation 10: The NHS AI Lab accounts for the environmental impact of the national medical imaging initiative and establishes how it aligns with a sustainable health and social care system

No international comparators had considered the environmental impact of their initiative or how they were positioned in relation to delivering a sustainable health and care system. This presents an opportunity for the United Kingdom to lead in this domain considering the health system needs not only for now but also for the future. We recommend that the NHS AI Lab develop an understanding of how the national medical imaging initiative could affect both positively and negatively an economically and environmentally sustainable health system. This is an important element of future-proofing the work and ensuring that it is fit for purpose in the coming decades (note: the NHS AI Lab strategy team has started considering how AI could contribute to the NHS goal of reaching net zero by 2045 and to an environmentally sustainable health and care system [53]).

The corresponding themes for this recommendation are (B) health system needs as the primary driver and (N) a focus on public and social good.

#### Policy and Regulation

Recommendation 11: The NHS AI Lab leverage its privileged position as the guiding health technology organization within both the civil service and the NHS to continue advocating and driving policy and regulatory change; the United Kingdom’s national medical imaging initiative is a tangible use case for uncovering the issues and providing examples of how they could be solved

All countries recognized that their current policies and regulations were not fit for the purpose of AI development and implementation in clinical settings. There was a range of mindsets regarding how to balance operating within constraints and advocating to change them. The NHS AI Lab is uniquely positioned within the government to drive the necessary changes in the United Kingdom making use of existing collaborations with regulatory bodies and DAs. We recommend that the national medical imaging initiative, with clearly articulated and demonstrable benefits to the health system, be used as evidence for this advocacy work.

The corresponding themes for this recommendation are (H) building on existing infrastructure and resources and (Q) advocating for policy, regulatory, and legal frameworks that are fit for purpose.

Recommendation 12: The NHS AI Lab leverage the work already undertaken in validation of AI models as a unique selling point for the United Kingdom’s national medical imaging initiative

No international comparators had progressed to the deployment and widespread adoption of AI-driven technologies developed through their initiatives. One of the bottlenecks for this is a clear validation process, an area in which the NHS AI Lab is well placed to take the lead given the existing work that has been done in this domain. We recommend that this is capitalized on as a unique selling point for the national medical imaging initiative to demonstrate an innovation funnel that runs smoothly through to the deployment of assured technologies.

The corresponding themes for this recommendation are (H) building on existing infrastructure and resources and (Q) advocating for policy, regulatory, and legal frameworks that are fit for purpose.

## Discussion

### Principal Findings

The NHS AI Lab sought to learn from countries developing medical imaging platforms to streamline the innovation-to-deployment journey for AI and ML algorithms for medical imaging. The research team conducted secondary and primary research with use cases from multiple countries to develop a deep understanding of the approaches for structuring a medical imaging platform program, how to set up supportive policy and regulatory initiatives, and form relationships with international stakeholders.

In addition to providing 12 recommendations for the NHS AI Lab to implement, the research team identified five areas in which the NHS AI Lab could offer a unique value proposition:

1. Galvanizing the already operating proof of concept, the NCCID program, to demonstrate benefit and secure stable United Kingdom government funding and support.
2. Within the new medical imaging platform, build in the ability to validate AI and ML algorithms as well as deploy them in health care settings. Only a few international initiatives built in the ability to validate algorithms and create a deployment pipeline, which is crucial for ensuring the effectiveness of algorithms during implementation.
3. Create wraparound offerings tailored to researchers, developers, and private companies operating in the United Kingdom. This may include tools to facilitate the creation of algorithms, training and workshops for upskilling, computational power, legal and regulatory support, and demand signaling for areas of clinical specialty in which there is high demand for AI and ML development.
4. Consider the environmental impact and sustainability of the medical imaging platform and the resultant carbon output from the outset.
5. Publicly demonstrate that the NHS AI Lab has incorporated collaborative international learnings and best practices.

### Strengths

The primary strength of the project was the NHS AI Lab’s openness to learning from other countries. Throughout our engagement with selected countries (Canada, Hong Kong, Japan, Singapore, Sweden, and the United States), we established that no other initiative had conducted international landscaping to inform strategy and implementation. Our work highlights the benefit of not reinventing the wheel in health AI initiatives but reaching out to build on the experience and expertise of others.

Second, the internal discovery team responsible for designing and building the NHS AI Lab’s medical imaging platform was engaged throughout the delivery of this project. Their engagement culminated in the workshop to elicit feedback and prioritize insights, followed by the selection of final recommendations. Often, policy and strategy research is conducted before or separately from the team creating and building a product. Policy and strategy research conducted in isolation may not provide practical and usable recommendations that can be taken forward during product development.

### Limitations

We identified 3 key limitations of this project. First, no literature review was conducted to inform the research. Owing to the novelty of creating medical imaging platforms for AI development, we instead decided to conduct a scan of potential international efforts via targeted Google, Google Scholar, and social media searches.

Second, the ICR reliability indicates some variation in coding assignments between the 2 researchers (KK and EP). Coding variability could be attributed to (1) the level of experience analyzing qualitative research and (2) the depth of understanding of the topics discussed by the interview participants. It is important to note that the resultant ICR of 0.41 indicates moderate reliability, which falls within tolerance as outlined by Landis and Koch [49] and O’Connor and Joffe [12].

Third, the study did not delve into the role and importance of postmarket monitoring or surveillance. In some interviews, it appears that this topic was not top of mind as they were working on initiatives that were in the beginning stages and algorithms were not yet actively deployed into the market for clinical use. However, since the completion of this project, the NHS AI Lab has funded the United Kingdom Medicines and Healthcare products Regulatory Agency to deliver several work packages, including updating legislation to require more robust postmarket surveillance for software as a medical device [54].

### Conclusions

Policy makers and digital developers internationally are chasing the potential for AI and ML algorithms to transform health care, with medical imaging seen as low-hanging fruit for realizing this ambition. Algorithms in health care are not confined to national borders, so how this ambition is realized by each country is particularly important. This paper outlines work undertaken by the NHS AI Lab to ensure that the investment in and creation of a generalized national medical imaging platform for the innovation and deployment of AI and ML algorithms in England is informed by international experience.

## Appendix

Multimedia Appendix 1 Template discussion guide.

Multimedia Appendix 2 Deductive thematic and coding framework.

Multimedia Appendix 3 Description of international initiatives.

Multimedia Appendix 4 Thematic synthesis.

## Abbreviations

AI: artificial intelligence
AIDA: Analytic Imaging Diagnostics Arena
DA: Devolved Administration
DHDP: Digital Health and Discovery Platform
GDHP: Global Digital Health Partnership
HADCL: Hospital Authority Data Collaboration Laboratory
ICR: intercoder reliability
MIDRC: Medical Imaging and Data Resource Center
ML: machine learning
NCCID: National COVID-19 Chest Imaging Database
NHS: National Health Service
PESTLE: Political, Economic, Social, Technological, Legal, and Environmental
TRE: trusted research environment
