# Supplementary material for: Learning From International Comparators of National Medical Imaging Initiatives for AI Development: Multiphase Qualitative Study
Source: JMIR AI. 2024 Jan 4;3:e51168. doi: 10.2196/51168 (PMC11041418; doi:10.2196/51168)
Supplement: Multimedia Appendix 1 [file ai_v3i1e51168_app1.docx]

## **Appendix I: Template discussion guide**

Interviews with prioritized countries were semi-structured covering contextual information, social and political reception, data handling, funding, commercialization, challenges and successes. The template discussion guide was adapted to the specific imaging platform identified for each country including validating any gaps or insights identified in the deep dive.

As the interviews were semi-structured, below are the primary questions, which are not an exhaustive list of questions asked of interview participants.

*Table 2. Semi-structured interview guide template*

| **Warm up questions** |
| --- |
| 1. Tell me briefly about your current role. |
| 1. What is your role and involvement in [*insert platform name*]? |
| **Contextual information** |
| 1. How would you describe [*insert platform name*] and its purpose?    - Who are the intended users of this platform?    - What modalities of imaging data does [*insert platform name*] include?    - Does [*insert platform name*] focus on any specific medical specialties?    - Within [*insert platform name*] do you link imaging data to other clinical data points? Why?   Prompts: Linked clinical data points might include health outcome data, the radiology report to accompany imaging study; What was the rationale/decision making behind linking other clinical data points to the medical imaging data   - - Do you share annotated images?   Prompt: for validation purposes, it’s necessary to be clear on where clinicians have identified pathology |
| 1. How was the idea for the project conceived?    - Who have been the biggest proponents driving it forward? |
| 1. Have you needed to enact any policy and/or regulation changes to set-up the initiative?    - Why? |
| **Social/ Political** |
| 1. How has the initiative been received by the public, health care professionals and wider stakeholders? |
| 1. What strategies are in place to maintain transparency and public trust? |
| 1. How have you ensured the impact of [*insert platform name*] does not worsen health inequalities?   Prompt: accessing to the research is equal, data is representative, improving health outcomes |
| **Data handling** |
| 1. How have the data and privacy laws in [country] shaped the architecture of [*insert platform name*]? |
| 1. What process is in place for collating the data for [*insert platform name*]?    - Who is the data controller?   Prompt: who has legal control over the data for those not familiar with the term data controller as official title |
| 1. What is the process for accessing data on the [*insert platform name*]*?*     - How did you come up with that process?    - Who is able to access the data?   Prompts: Do you have to have a specific research project? Do you have to have a certain academic affiliation? Can businesses access it?   - - Under what terms is the data accessible?   Prompst: Can it be accessed remotely? Is it stored in a physical place? |
| 1. What are the incentives for institutions and stakeholders to participate in [*insert platform name*]? |
| **Funding, Commercialization and IP** |
| 1. What is the funding model for [*insert platform name*]?    - Where is the funding coming from?    - How is that funding being disbursed? |
| 1. What is the commercial model?    - Is the intent to recoup cost or profit?    - If for profit, how would this be redistributed amongst contributors?   Prompt: If at appropriate stage of commercial maturity or if the issue arises, can ask further about legal status and ability to own shares and IP. |
| 1. What is the IP model for products developed using the platform?   Prompt: this will inform how countries and health systems get to use the innovations that are developed - consider distinction between background IP i.e. that has been generated by setting up the platform itself, versus new IP i.e. developed using the platform |
| **Challenges, lessons, and success** |
| 1. Have any AI products or solutions products successfully made it to clinical deployment due to [*insert platform nam*e]?   Prompt: Why/why not? |
| 1. What impact (positive/negative) has the initiative had on the surrounding landscape? |
| 1. Has the project faced any opposition or challenges?   Prompt: Describe opposition faced i.e., financial, stakeholders, regulatory, social, technical |
| 1. What’s gone well with setting up and developing [*insert platform name*]? |
| 1. What would you do differently if you could do it again? |
| **Environmental** |
| 1. What consideration has been given to the sustainability of this work?    - In terms of funding and impact from an environmental perspective |

### 
