# Supplementary material for: Learning From International Comparators of National Medical Imaging Initiatives for AI Development: Multiphase Qualitative Study
Source: JMIR AI. 2024 Jan 4;3:e51168. doi: 10.2196/51168 (PMC11041418; doi:10.2196/51168)
Supplement: Multimedia Appendix 2 [file ai_v3i1e51168_app2.docx]

## **Appendix II: Deductive thematic and coding framework**

Deductive themes were structured around the PESTLE analysis framework applied to the desk research, and the interview guide. These were differentiated into subthemes with corresponding codes and subcodes to mark up the transcribed interviews.

*Table 3. Deductive thematic and coding framework for semi-structured interview analysis*

| **Theme/Sub-theme** | **Code/sub-code** | **Definition** |
| --- | --- | --- |
| **1. Context** | | |
| 1.1 Purpose of the platform | 1.1 Purpose | What the initiative (platform and wrap around service offering including products, features, and additional activities) is aiming to achieve (future and present) and products, features and additional activities offered. |
| 1.2 Intended users of platform | 1.2 End users | The intended and/or actual users of the initiative. |
| 1.3 Medical Specialties of interest | 1.3 Specialties | What specialties and/or areas of medical interest the data primarily relates to (differentiated from linked data). |
| 1.4 Data modalities | 1.4 Modalities | The types of primary imaging and/or other data the platform collects e.g. imaging modality (radiology, histopathology, scope imaging, retinal imaging), surrounding metadata, imaging reports. |
| 1.5 Annotations | 1.5 Annotations | Information relating to if images are annotated, the rationale for this, and how annotated images are used. |
| 1.6 Associated clinical data points | 1.6 Linked data | The types of secondary data the initiative collects e.g. comorbidities, demographics, social determinants of health. |
| **2. Political** | | |
| 2.1 How project was conceived | 2.1 Inception | Reason for the initiative being established and actors involved in this process. Where, why and how the idea originated, and the context in which this happened. |
| 2.2 Biggest proponents of the work | 2.2 Proponents | The biggest proponents (individual and/or organization) of the platform and/or initiative that led to the platform creation. |
| 2.3 Level of government support | 2.3 Political will | Demonstrable government support in the form of funding, explicit endorsement of the work (either public or private statement), level of collaboration in tackling legal/policy/regulatory barriers. |
| 2.4 Number of involved institutions | 2.4 Scale | The number of institutions involved in the conception, set-up (inc. data processing), and implementation of the initiative, including those receiving and/or using service offerings. |
| 2.5 Statutory bodies and oversight | 2.5 Governance | How the initiative and participating organizations are managed and overseen in the wider context of the health system, with reference to relevant statutory bodies. |
| 2.6 Health and care structure | 2.6 Health system | The country’s existing and influential health system and care structures and principles. |
| **3. Economic** | | |
| 3.1 Public/private sector stakeholders | 3.1 Stakeholders | The nature of involved institutions, including (a) those who have funded/contributed to establishment and ongoing running of the initiative, (b) those who are providing data and services, and (c) those who are using the initiative |
| 3.2 Commercial model/plan | 3.2 Commercial | The current and potential commercial model and viability of the initiative. Commercial models could include for profit, not for profit. |
| 3.3 Funding source and disbursement | 3.3 Funding | How the initiative was funded, where this funding has come from and over what time period, conditions attached to the funding, and if/how funding is disbursed within the initiative. |
| 3.4 IP model/plans | 3.4 IP | The current and potential IP models for AI algorithms and/or data developed and/or consumed in the platform |
| **4. Social** | | |
| 4.1 Views on project/AI/data (public) | 4.1 Public attitudes | Reference to public attitudes and perceptions of (a) the initiative itself, (b) development and use of AI, and (c) use of medical and/or personal data. |
| 4.2 Views on project/AI/data (prof.) | 4.2 Professional attitudes | Reference to professional attitudes and perceptions of (a) the initiative itself, (b) development and use of AI, and (c) use of medical and/or personal data. Professionals could range from healthcare practitioners, media, policy makers and technologists. |
| 4.3 Strategies for engagement | 4.3 Engagement strategy | Action taken to (a) involve stakeholders and users design and develop of the initiative, (b) encouraging active engagement from intended users, and (c) engaging the wider public and professionals in the initiative to build trust |
| 4.4 Maintaining transparency and trust | 4.4 Trust | Action taken to maintain transparency and trust with the public and professionals (which can include stakeholders and users of the initiative), or specific challenges that fall under this domain. |
| 4.5 Impact and health inequalities | 4.5 Inequalities | Consideration given to (a) representativeness of data collected, (b) mitigating bias, (c) equity of access to platform and knowledge, (d) equality of benefiting of outputs from platform. |
| **5. Technological** | | |
| 5.1 Platform infrastructure | 5.1 Infrastructure | The technical infrastructure including overarching structures, products and features contained within the platform and/or and wrap around service offering including products, features, and additional activities. |
| 5.2 Have products made it to market | 5.2 Product deployment | Whether AI products have made it to market; relevant stages of this/projected timelines; specific considerations given to this process (even if hypothetical and nothing has been deployed) |
| **6. Legal** | | |
| 6.1 Impact of data and privacy laws | 6.1 Legal | The impact of existing or historical data and privacy laws on the inception and shape of the initiative and/or platform, and approaches taken to navigate relevant laws. Country specific information about national data, IP, and privacy laws. |
| 6.2 Process for data collation/control | 6.2 Data handling | The process for identifying, collating, handling (inc. deidentification) and making of data accessible within the platform. This may include additional non-data specific tasks required to make data available such as data controller/receiver arrangements. |
| 6.3 Process for accessing data | 6.3 Data access | The process for allowing users of the platform to gain access to relevant data including how data requests are handled, how data is deemed relevant per request, how data is shared with users, and how data is disposed of. |
| 6.4 Incentives for participation | 6.4 Incentive | How and why users and wider stakeholders are incentivized to participate in the initiative including providing funding, support, and sharing of data. |
| 6.5 Policy considerations | 6.5 Policy | Information about the policy context (historical, current, and future) that the initiative operates in, including impact on its shape and implementation. Additionally, any outstanding policy questions raised by the initiative (active, passive). |
| 6.6 Regulatory considerations | 6.6 Regulation | Information about the regulatory context (historical, current, and future) that the initiative operates in, including impact on its shape and implementation. Additionally, any outstanding regulatory questions raised by the initiative (active, passive). |
| **7. Environmental** | | |
| 7.1 Sustainability considerations | 7.1 Sustainability | Any consideration given to immediate and/or long-term environmental impact. |
| **8. Insights** | | |
| 8.1 Impact on surrounding landscape | 8.1 Impact | If/how the initiative has influenced the wider landscape, be that across policy, regulation, health and care and/or across data and AI application in other sectors. |
| 8.2 Opposition and barriers | 8.2 Challenge | Specific barriers or opposition that occurred. |
| 8.3 Things that have gone well | 8.3 Success | Specific facilitators or activities that have occurred. |
| 8.4 Things to do differently | 8.4 Lesson | Reflections on what could be done better or differently. |

##

### 
