# Supplementary material for: Learning From International Comparators of National Medical Imaging Initiatives for AI Development: Multiphase Qualitative Study
Source: JMIR AI. 2024 Jan 4;3:e51168. doi: 10.2196/51168 (PMC11041418; doi:10.2196/51168)
Supplement: Multimedia Appendix 3 [file ai_v3i1e51168_app3.docx]

## **Appendix III: Description of international initiatives**

Detailed overview of each country's medical imaging initiative for AI/ML based on findings from PESTLE analysis and semi-structured interviews. The summary provides learnings on the initiative's background, key stakeholders, funding sources, approach to commercialization, and triumphs and challenges.

*Table 4. Overview of Canada’s DHDP*

| **Background** | **Stakeholders** | **Funding** |
| --- | --- | --- |
| - Canada launched their New Digital Charter (2019) to guide Canada in building an innovative, people-centered and inclusive digital and data economy - Canada was the first country in the world to adopt a National AI Strategy with federal investment - Diverse data sources (e.g., genomics, imaging, electronic medical records) will be integrated in the DHDP to deliver precision medicine therapies - DHDP clinical condition of interest is cancer, with other conditions being introduced over time - The DHDP’s intended federated data ecosystem reflects Canada’s stringent attitudes towards data privacy and sharing | - The Imagia and the Terry Fox Research Institute are developing the DHDP with support from the Canadian government - The DHDP has over 97 consortium partners, spanning academia and the private sector, including hospitals, pharma, medical device companies, SMEs, etc. | - In the past five years, funding for Canadian AI companies has increased year-on-year, with Toronto start-ups raising over CA$1B each quarter (2021) [16] - The DHDP received an initial CA$49M through Innovation, Science and Economic Development Canada's Strategic Innovation Fund (SIF) [17] - The DHDP received additional funding of over CA$159M and in-kind contributions from the 97 partners |
| **Commercialization** | **Triumphs** | **Challenges** |
| - Commercialization is of the discoveries made through the DHDP, not the medical data used in the platform - The initiative will help commercialize “homegrown” Canadian innovation - The DHDP team is encouraging active participation of big pharmaceutical and biotechnology companies, not just financial contributions - “Allocentric” approach, where the commercial benefit is shared according to the contribution of each organization | - DHDP has financial support and sustainability secured for the foreseeable future from both public and private sources - Broad stakeholder buy-in and political will from the Canadian federal government for the DHDP to succeed and provide a model for future digital innovation | - Canada’s policy and regulation for AI was not fit for purpose, discordant with technology and expertise - The implication of existing and new personal information and data privacy regulations, such as the adoption of the General Data Protection Regulations (GDPR) in the European Union (EU), putting pressure on Canada to follow suit - Convening the breadth of expertise required for successful AI-driven technologies |

*Table 5. Overview of China’s National Medical Image Database*

| **Background** | **Stakeholders** | **Funding** |
| --- | --- | --- |
| - The Chinese government wants China to become a world-leading AI innovation center by 2030 [18] - The Chinese National Medical Image Database was approved by the National Health Commission [19] - The public in China has the greatest levels of acceptance of AI [20] and more trust in private companies handling personal information [21] in comparison to other countries | - The Chinese Society of Radiology is building the database - The venture will ultimately involve 350-400 hospitals | - In 2018, the market size of AI was CNY20B [22] - No public disclosure of specific initiative funding |
| **Commercialization** | **Triumphs** | **Challenges** |
| - While experiencing investment, it was predicted most Chinese AI start-ups could fail amid fierce competition, pressure to commercialize, and wider economic shutdown [22] | - Reforms of the Chinese health system have brought increased public financing and improved access to healthcare [23] - Government push and promotion of AI technologies in healthcare | - Unclear what technology stack the Chinese National Imaging Database will employ and how the initiative will overcome issues of data digitization, cybersecurity, and commercialization - The use of electronic medical records is unstandardized and underdeveloped, with policies from the National Health Commission to encourage usage [22] - Cybersecurity law means a lot of health data is subject to strict localization requirements and security reviews [22] |

*Table 6. Overview of Hong Kong’s HADCL*

| **Background** | **Stakeholders** | **Funding** |
| --- | --- | --- |
| - Hong Kong has strong government support for and promotion of using AI technology in healthcare and other sectors [24-25] - HA had a political mandate in 2017 to open their extensive, longitudinal health data set with over 20 years of records for big data analytics - HADCL contains over 280 terabytes of clinical data from HA, including demographic, admissions, diagnostic, medication, clinical, and radiology data - A recent survey shows majority support by the public for applications of AI to health and care | - HADCL initiative is built, owned, and led by the HA with support from the Hong Kong Government - At the time of research, the HADCL was only open to universities, not to commercial actors, but the HA was exploring opening the platform for commercial actors in the future | - In 2018, the HADCL initiative received a One-off injection of government funding (amount not disclosed) - The HADCL continues to receive annual CapEx funding from the government - The HA is looking for new funding routes as the HADCL progresses |
| **Commercialization** | **Triumphs** | **Challenges** |
| - Hong Kong is a unique market to enter, as it is geographically small and provides a sizeable population to pilot new initiatives with [26-27] - To access the HADCL applications go through an approval process and, if approved, data access is then provided for free. - The contract includes IP arrangements to grant HA perpetual usage license for resulting products developed with the data | - A vast health data repository managed using a single clinical management system - The HA has inhouse health informatics expertise and upskilling programs (e.g., internship programs) - The HADCL self-service data platform offers samples for proof-of-concept | - At the time of research, Hong Kong had no specific AI laws or regulations - The HADCL data has to be accessed on- site only within working hours due to stringent security. The HA is exploring options for providing remote access |

*Table 7. Overview of Japan’s Research Centre for Medical Big Data*

| **Background** | **Stakeholders** | **Funding** |
| --- | --- | --- |
| - The health technology science sector in Japan has lagged compared to other industries due to conservative ideas about health and high baseline services, resulting in less imperative or urgency to pursue technological solutions [28] - Japan’s Fifth Science and Technology Basic Plan (2016) promoted Big Data, AI and Internet of Things [29] - However, public awareness and support for digital technology are unclear and are a barrier to achieving government ambition [30] | - Led by the National Institute for Informatics (NII) - The Research Centre for Medical Big Data sources medical images through academic societies (>10 million medical images, 2019), including Japan Radiological Society, Japanese Ophthalmological Society, Japan Gastroenterological Endoscopy Society - The initiative also leverages the Science Information Network 5 (SINET5); a ultra-high-speed Japan-wide network connecting >800 academic institutions - Users of the platform are academic institutions | - The health tech industry is forecasted to reach 225B yen by 2025 [31] and is identified as a potential area to boost Japan’s recently stagnant economy [32] - Funded by Japan Agency for Medical Research and Development (AMED); however, the value of the funding was not undisclosed - At the time of research, no funding had been secured for the financial year 2021/2022 |
| **Commercialization** | **Triumphs** | **Challenges** |
| - No clear commercialization strategy - Complex data-sharing arrangements and patent/IP agreements made it difficult to establish a viable commercial model - Data sets are only accessible to non-commercial actors | - Relatively advanced regulatory workflows for AI as a medical device - 2017 medical big data law allows for the pooling of medical records (if anonymized) for research purposes | - Lack of financial sustainably made the future of the Research Centre for Medical Big Data uncertain - Data pipelines were an initial challenge, addressed by working with medical societies - NII was unclear on how to return benefits to the taxpayer |

*Table 8. Overview of Singapore’s AI-enabled Medical Imaging Platform*

| **Background** | **Stakeholders** | **Funding** |
| --- | --- | --- |
| - In 2019, Singapore launched its National AI strategy [33] - The AI-enabled medical imaging platform is a use case for cross-sector learning - The platform is starting with imaging data and hoping to expand to other health data modalities - The team wants to facilitate data-sharing and address the challenge of deploying resulting technologies at scale | - Led by the Integrated Health Information System (IHiS) Health Lab, alongside other undisclosed public and private collaborators - IHiS provides IT and technology services to all public healthcare settings in Singapore | - Funded by the Ministry of Health - The initial amount and duration of funding are not publicly disclosed - If the IHiS Health Lab can prove successful with the first tranche of funding, they will apply for a second |
| **Commercialization** | **Triumphs** | **Challenges** |
| - No details yet on commercial arrangements data-sharing, but it seems to operate primarily on a case-by-case basis for all data-sharing requests - The suggestion that users will pay a subscription fee to access data - Singapore's government has previously collaborated with major corporations on big data projects (e.g., Apple), so there is a precedent for these partnerships | - Strong political will, as the National AI Strategy has an emphasis on cross-sector collaboration and public benefits from initiatives are likely to be shared beyond health and care | - Acknowledgment of ubiquitous challenges across the lifecycle of AI-driven technologies with reference to the regulatory landscape - Challenges around how to ensure the security of the platform and patient data |

*Table 9. Overview of Sweden’s AIDA*

| **Background** | **Stakeholders** | **Funding** |
| --- | --- | --- |
| - The Swedish Government’s *National Approach to AI* (2018) has limited references to health [34]. However, the *Vision for E-Health 2025* (2020-2022) sets a national goal for Sweden to be a leader in utilizing digitization and e-health opportunities by 2025 [35] - In 2018, Sweden adopted the Data Protection Act [36] - Past mandated creation of national registries containing >5 terabytes of health data provided the foundation for AIDA | - AIDA is part of the National Strategic Innovation Program Medtech4Health, a joint initiative by VINNOVA, Formas, and the Swedish Energy Agency [37] - AIDA is hosted by the Center for Medical Image Science and Visualization (CMIV) at Link**ö**ping University - The initiative has around 40 formal partner organizations and over 60 other participating organizations | - Investment in Swedish health tech companies reached €409m in 2020 (the highest in several years) [38] - The initiative is funded by the Swedish Government Innovation Agency, Vinnova, via the strategic innovation program Medtech4Health - AIDA disburses this funding to projects, as well as the AIDA infrastructure being fully funded - The total funding amount is not publicly disclosed - The participating projects are responsible for 50% of costs, which is matched by 50% funding from Vinnova - AIDA’s computational system is fully funded as a common resource |
| **Commercialization** | **Triumphs** | **Challenges** |
| - AIDA is a not-for-profit initiative - Corporate partners include Siemens and Sectra, and it was unclear how these corporate relationships align with the not-for-profit model | - Sweden has a strong national IT and tech infrastructure, digital literacy and maturity [39] - AIDA’s initiative emphasizes community building, which creates a strong narrative purpose - At the time of research, at least 25 innovation projects were already up and running | - Getting broad support for a field (AI technology) where there is a lot of hype and misunderstanding - Difficulty obtaining other data modalities beyond imaging - Sweden’s Patient Data Act currently limits access to large amounts of patient data for purposes other than research project [40] - Navigating copyright and patenting: copyright can be applied to AI algorithms if they are considered to be a computer program and a patent cannot be applied to an algorithm because it’s a mathematical model [39] |

*Table 10. Overview of USA’s MIDRC*

| **Background** | **Stakeholders** | **Funding** |
| --- | --- | --- |
| - The US has favorable federal government policies for AI, including R&D investment and national AI strategy [41] - 84% of US radiology clinics had adopted or planned to adopt AI programs [42] - MIDRC is an open-access platform to collect, annotate, store, and share COVID-19 medical images (with a view of expanding to other areas) hosted by the University of Chicago [43] - COVID-19 is described as a serendipitous use case to galvanize existing efforts toward creating an imaging data commons | - Initiated by National Institute of Biomedical Imaging and Bioengineering (NIBIB), part of National Institutes for Health (NIH) - Collaborators include the American College of Radiology, the Radiological Society of North America, and the American Association of Physicists and Medicine | - The US market for AI in health is projected to grow from US$1.7bn (2019) to US$32bn (2027) [44] - Funded by NIBIB emergency COVID-19 fund for an estimated $20M over a 1-2 year period with further funding contingent on performance [43] - Financial sustainability of initiative unclear beyond pandemic funding |
| **Commercialization** | **Triumphs** | **Challenges** |
| - Initiative reluctant to explore commercial options but is aware that the need may arise to make sustainable - Instead, the initiative is focused on being a “public good” and offering data commons - At the time of research, no IP-sharing agreements were in place | - FDA regulation for AI-driven technologies is fairly advanced - Able to leverage pre-existing infrastructure and data pipelines - Active steps to ensure data is representative of the population | - At the time of research, agreements for sharing the relevant medical imaging data were in the process of being signed with several sites, but no data was being hosted on the platform - Navigating federal law and managing administrative overheads |

### 
