# Supplementary material for: Learning From International Comparators of National Medical Imaging Initiatives for AI Development: Multiphase Qualitative Study
Source: JMIR AI. 2024 Jan 4;3:e51168. doi: 10.2196/51168 (PMC11041418; doi:10.2196/51168)
Supplement: Multimedia Appendix 4 [file ai_v3i1e51168_app4.docx]

# Multimedia Appendix 4: Thematic synthesis

Thematic synthesis of learnings about other countries’ initiatives from phase 3 and 4. The thematic synthesis is broken down into primary and secondary categories.

| Category and subcategory | | Thematic synthesis |
| --- | --- | --- |
| **Purpose** | | |
|  | A. Demonstrable benefit of the national medical imaging initiative | - Some countries had or were starting to prove demonstrable benefit of their imaging initiative at the onset to obtain buy-in from stakeholders and the public. It is worth noting that demonstrable benefit of a country’s initiative did not guarantee enduring government funding. - Canada and Singapore aspired to demonstrate benefit through proofs of concept and targeting identified areas of need within the health system. Canada, for example, had already deployed their platform in 5 hospitals—this was important for them as they wanted to deploy internationally but first had to test out the infrastructure approach within the country. - In contrast, Sweden made no specific reference to proving benefit, Japan had a test case in histopathology imaging but did not link this to demonstrating benefit, and the US initiative considered itself at too early a stage to deliver any demonstrable benefit. Hong Kong, at the time of the study, had no deployment or proof of concept. They spoke about less tangible or quantifiable benefits (eg, positive reception and embracing innovation). Hong Kong had developed assessment criteria for understanding the impact of technologies, which included criteria that the Hospital Authority will benefit from any algorithms developed and the public good. - When thinking about who the end beneficiary of the initiatives would be, for most countries, it was patients and health care professionals, researchers, or funding bodies. Singapore, Sweden, Canada, and the United States explicitly mentioned delivering benefits to patients and communities and helping patients understand how AI^a^ can positively affect their lives over time. - The workshop participants reflected that demonstrating benefit early would enable countries to make their initiative into a tangible object or use case rather than a hypothetical value proposition that may be hard for stakeholders or the public to engage with. Workshop participants highlighted the importance of a clear narrative:   - “There are aspects there [of the purpose] where [Singapore] got those stepping stones, where they’re absolutely clear on why. For our initiative, we obviously want to get to a really logical, rounded view of what this service or platform means to us, why we’re doing it, what it’s addressing, in what order we’re going to develop that story.” - Workshop participants were also vocal about fears regarding lack of demonstrable benefit when reflecting on the initiatives presented:   - “For some, there’s no real benefit...it’s all ‘let’s wait and see.’ There’s lots of time and investment that’s gone into this with individuals driving it because it’s their interest project...” - The expectation of being able to provide demonstrable benefit should be caveated at different stages of development. In the case of the United States, for example, the imaging initiative is still in setup stages, and no data have made it to the platform. The United States’ ability to demonstrate the benefit of the imaging initiative, which will be a public database of COVID-19 chest images, remains hypothetical. The initiative organizers are not involved in the end-to-end deployment pipeline. Although the platform data will be made public, at the time of the study, a strategy on how and why those data are used in response to the pandemic was still in progress. |
|  | B. Health system needs as the primary driver for AI development | - There was consensus across the countries that the development of AI models and AI-driven technologies should be based on a health system or user need. In some cases, the system-wide nature of these needs was reflected in how the initiative was organized. The Canadian DHDP^b^ was organized into “the science stack, policy stack and technology stack” to ensure that strategic decisions and resources were made taking a holistic view of where and if AI would be appropriate. - Other drivers of purpose and initiative design were identified that may either align with or diverge from health system needs. These included personal interest (Canada), political will (Hong Kong), and feasibility within legal constraints (Canada). - In some instances, the drivers were not clear to those unfamiliar with extensive detail of the initiatives. Although this may have been a limitation of the workshop facilitation, it might also suggest a need for clearer communication and engagement from the initiative itself, especially regarding if and how it would successfully commercialize and financially sustain itself beyond initial funding. - Workshop participants raised the counterargument that focusing on need could hinder innovation:   - “How much of a problem is the fact that it’s bound to existing healthcare challenges...Obviously that’s where you want to prove the benefit...but it’s a barrier in terms of the pace of what you can be innovating and experimenting with.” |
|  | C. Community and shared purpose | - For most countries, ideas regarding community and shared purpose were reflected in the framing of the initiative. Interdisciplinary work was also highlighted as a priority by Sweden, which recognized that this would aid the development and deployment of AI models in clinical practice. The perceived benefits of fostering a community included widening participation with the initiative, pooling resources to allow for activities that would otherwise be unfeasible, and sharing of knowledge and mindsets:   - “It’s been supported both from the grassroots side of things and we’ve listened to ‘what do you need?’” [Interview participant; Sweden]   - “It’s intended to be open for everyone. It’s intended to be publicly accessible data. Safe harbor, fully de-identified.” [Interview participant; United States] - Workshop participants pointed to the importance of shared purpose and narrative for bringing people and mindsets together. However, workshop participants acknowledged that there was a tension that might arise between community building and commercialization:   - “To frame this as a meeting place over a marketplace...as soon as you say market place it feels very transactional, commercial orientated.” - Workshop participants pointed out that the involvement of the Devolved Nations (Wales, Scotland, and Northern Ireland) presented a unique community-building challenge in the United Kingdom. |
| **Users** | | |
|  | D. Embracing and enabling the central role of health care professionals to design and develop AI-driven technologies | - Almost all countries’ targeted user base for their initiative was health care professionals, especially clinicians working in clinical practice:   - “One of the goals of the platform is really to empower the clinicians so that they can make the discovery on their own, at their own pace, on their own data even if they’re not AI experts.” [Interview participant; Canada] - However, in this context, it was apparent that “healthcare professionals” creating AI products was not synonymous with people who understand the need in the health system:   - “A lot of researchers don’t really have that proximity to actual clinical reality so they can get surprised when they try to deploy their models with very nice performance figures and they go out to the clinic and see it doesn’t work, or it doesn’t fit into the workflow.” [Interview participant; Sweden] - All countries recognized that AI-driven technologies should be informed and shaped by health care professionals. The countries were taking steps to intimately involve health care professionals in AI design and development by creating wraparound services and easy-to-use tools to fill the knowledge and technical skills gap that health care professionals have when it comes to AI. In Sweden, for example, stakeholders can join without defined project ideas as a “network partner” and take part in the knowledge exchange and shared data sources within AIDA^c^. Sweden also offers clinical fellowships, which encompass continued education while health care professionals work on a specific project. - To facilitate participation of nontechnical users (who were more often than not a type of health care professional), some countries took steps to (1) remove the need for technical upskilling or (2) provide targeted upskilling support. Some countries removed the need for health care professionals to technically upskill by providing custom AI tools or human services to plug technical gaps as part of the initiative wraparound offering:   - “We do offer an array of tools, and if a researcher has a particular software tool they want to use we’ll actually usually provide that. We also have health informatics experts.” [Interview participant; Hong Kong] - In other instances, upskilling support was offered in the form of training. Sweden provides training courses for health care professionals, and Hong Kong develops the next generation of expertise through internships and research posts:   - “Very popular three-day AI course for radiologists and pathologists coming and actually doing some hands-on AI development as well.” [Interview participant; Sweden] - Workshop participants agreed with the importance of health care professional participation, acknowledging that the ease of this may depend on the overarching context of the initiative, what type of organization is leading it, and whether the initiative is primarily angled toward academia or not. The workshop participants clarified that they were not making an assumption that health care professional participation would be more difficult outside an academic context but rather highlighting that equivalent means were needed to allow for and encourage their involvement and this should be factored into the initiative design. |
|  | E. Recognizing that users are not discrete groups | - Most countries touted that their initiatives were “open to all”; however, this often still referred only to those from certain sectors, such as academic or government-affiliated researchers. Initiative leads were cognizant of the existing ties and relationships between user groups (ie, academia, government, pharmaceutical and data science companies) and how these might play out within the initiative. In Japan, for example, the platform was an “open platform” for researchers affiliated with universities without encouraging use by industry-affiliated academics. However, the initiative leads recognize that academic researchers sometimes have commercial ties and interests that they may not be aware of. - Conflicts of interest were particularly pertinent in instances in which industry players had significant involvement in the running of the platform, which was recognized as a challenge for the NHS^d^ AI Lab. Some of the fears voiced by workshop participants suggested a perceived (and perhaps artificial) division between academics and commercialization interest or deployment capacity. By recognizing that users are not always discrete groups, the workshop participants believed that they would be able to tailor their offerings in the most applicable ways. This requires avoiding assumptions about who plays what role in the process and not pigeonholing users’ needs. |
| **Organizational** | | |
|  | F. Importance of wraparound service offerings | - Discussion from the workshop underscored consensus on the importance of service offerings beyond the data platform itself and what a strong incentive this was for user participation. Wraparound services broaden participation by providing scope for upskilling, if desired, and removing the need to upskill if not. Offerings include software, human resources of expertise, computational power, education, and support with navigating legal and regulatory barriers. Sweden, for example, provides a “computation system that is a 16 GPU system that is a very nice piece of equipment that is much too big and expensive for any individual research group in Sweden to buy or work with” [Interview participant; Sweden]. - Hong Kong highlighted the importance of not only providing these wraparound services but clearly communicating to users what is on offer and how this dovetails with their aspirations. The Hong Kong stakeholders spent time developing and offering a suite of supportive trainings for users of their platform:   - “We run several kinds of workshops so that researchers are fully aware of the data, the possibilities and we will talk to them about what they’re trying to achieve.” [Interview participant; Hong Kong] - Wraparound services such as those offered in Sweden and Hong Kong contribute to community building by facilitating interdisciplinary work and learning. The workshop participants recognized the importance of identifying the right wraparound services for users of the NHS AI Lab’s platform. |
|  | G. Tempering the pace of development | - All countries highlighted the pressure to deliver rapid results and the need to balance this with considerations regarding the sustainability of their initiative and best practices. Pressures to develop at pace included political factors, funding arrangements, and fulfilling unmet needs, especially in cases in which the COVID-19 pandemic was a major driver. - Political will for the initiatives manifested in galvanizing cooperation (Canada), approval won for moving quickly (Japan), and injections of government funding to set up the initiative at pace (United States). The countries also noted how high-profile government announcements, launch of national AI strategies, and election cycles had shaped the support for the initiatives. - Not all initiatives moved at such a pace. For example, the trajectory of Hong Kong’s initiative has been less steep, allowing them to build logically toward a long-term vision over 25 years. The workshop participants reflected on the holistic nature of the Hong Kong initiative compared with those of other countries and the step-by-step achievements as they built out the initiative. Workshop participants acknowledged the temptation to “speed ahead” with the NHS AI Lab national medical imaging initiative without considering the repercussions of doing so. |
|  | H. Building on existing infrastructure and resources | - Japan, Singapore, Hong Kong, Sweden, and the United States acknowledged that the initiatives were only made possible by virtue of existing infrastructure and resources. Singapore, for example, was considering leveraging the existing private cloud infrastructure that hosts medical records as their secure environment and stressed the importance of aligning new projects with existing technical infrastructure. Hong Kong pointed to existing data pipelines as a cornerstone of the work, whereas the United States acknowledged that its initiative would not have been possible without historic investment. The Swedish initiative benefited from existing collaboration between the lead research group and industry partner Sectra. - Although necessary, existing elements were not sufficient to support the initiatives, and they all relied on an up-front and potentially continuous injection of government funding and support. This returns to the challenge of financial sustainability. None of the countries have entirely solved this for their initiatives, but some are in a far better position than others. Hong Kong, for example, is likely to receive ongoing government funding for their 2-decade–long project, whereas Canada has prioritized developing a strong commercial model from the outset. |
| **Commercialization** | | |
|  | I. Ensuring financial sustainability | - Most countries raised concerns regarding the financial sustainability of the initiatives having been founded on one-off injections of government funding. There appeared to often be conflict between the inherent value of an initiative (for open access or public good) and pragmatism regarding how to fund it in the long term without relying on injections of government funds (implied to be hard won and uncertain). - In the United States, for example, the national imaging initiative is focused on COVID-19, with people engaging to share data for the public good:   - “We’re not naïve to commercialization. I’ve heard people talking about that, but I think the goal is to provide this as a public good and not to try to commercialize it.” [Interview participant; United States] - This conflict suggested an assumed division that public goods cannot be commercialized or that, in doing so, the ethos of the initiative would be undermined. However, it was acknowledged that financial sustainability would at some point have to pragmatically take precedence. - Although the United States team appeared to not be seeking to commercialize the platform, the initiative’s long-term financial sustainability is in question. Even if the project team wanted to create a sustainable commercial strategy, the way the United States initiative is set up means that they are unable to capitalize on the data held within the platform and do not hold IP^e^ rights for products developed using the platform. - In contrast, Canada, having the most commercialized model, was least concerned about financial sustainability. The initiative was set up with both public and private investment and encouraged private investment and collaboration to shape and grow the direction. |
|  | J. Differing or absent commercial approaches | - Canada appeared to be the only country considering commercialization of their national medical imaging initiative up front and as part of the initiative’s organizational model. Other countries, although they were starting to think about commercialization, stressed that this was not a priority (even with doubtful future funding) and that their initiative was for the public good:   - “How to get the profit from the industry is...maybe we should do it afterwards, that’s my policy.” [Interview participant; Japan] - As discussed, previously, Japan and the United States expressed that their hands were tied when it came to the commercialization of the initiatives because of the funding model, data and privacy laws, and their data-sharing agreements. Some countries were considering different ways to commercialize aspects of their initiatives (ie, validating algorithms), recognizing that it does not have to be all or nothing. - The workshop also revealed negative connotations associated with commercialization:   - “The Hong Kong initiative seems to be very research bent by academics deploying AI, which I think then takes away a load of that commercialization problem because everyone’s doing stuff for clear motives, for a greater understanding...there’s no ulterior motives you need to flush out.” [Workshop participant] - Workshop participants also shared concerns that building commercialization into the United Kingdom’s national medical imaging initiative too early might harm innovation:   - “For us, because we want to encourage innovation, but we don’t know what we want that innovation to be yet, I’m concerned that if we start looking for too many commercial models now we’re going to get pulled in the wrong direction.” [Workshop participant] - Similar to the workshop participants, countries appeared reluctant to frontload thinking about commercialization—the conversations conveyed the sense that commercialization was held in opposition to delivering a public good, and many countries seemed hesitant to make a perceived compromise on values by commercializing their initiative. There was a cognitive dissonance between deprioritizing commercialization and simultaneously recognizing the lack of financial sustainability as the greatest threat to the longevity of the initiatives. By not considering commercial avenues early in development, countries ran the risk of inadvertently excluding commercial options by designing the initiative (especially data-sharing arrangements) without this in mind. |
|  | K. Common and continuing data challenges | - All countries recognized, to varying degrees, the challenges associated with having health data for AI development, including accessing data, cleaning data, and having data to test and validate algorithms. Most countries touched on the need for representative data; however, only 2 countries (the United States and Sweden) shared how they had designed data collection to ensure that it was representative of the population. - Some countries were not selective with data sets for training and validation, which could lead to bias or lack of population representation between the sets:   - “We don’t make a clear distinction between training data and validation data. We just asked for that annotated data and on our side we divide them into test data and training data and do the research.” [Interview participant; Japan] - Most countries had designed their initiatives to mitigate data security concerns and acknowledged that public trust (or lack thereof) contributed to this decision. The Canadian platform, for instance, was going to be a federated platform so that the data stayed held in the individual institution and only the resultant algorithm was shared. In line with Canada’s stringent attitudes toward data privacy and sharing, the project team used a “privacy by design” ethos for their strategic and technical decisions. |
|  | L. Subsetting data offerings | - Some countries subset data offerings to users’ needs from the outset. Hong Kong worked closely with users to fulfill their data requirements by running a proof of concept and supporting the researchers to identify the right data for their needs. - Many countries spoke about the desire to expand the breadth of data offered and extend the focus of the platform beyond its initial purpose. The United States wanted to expand the platform from collecting medical images to also collating clinical and demographic information about the patient. Singapore had ambitions to extend the data collected into the “social data space,” such as data from wearables (ie, steps taken, exercise levels, and hours of sleep) and self-reported measures (ie, food eaten today). - Sweden took the unique approach of creating DOIs^f^ for data sets to support IP and validate an academic’s contribution to data sets and associated research conducted using that data set:   - “We have the ability to create our own digital object identifiers for the datasets...They can be cited as datasets like any academic article...‘If you want to use my data, you can cite my data as such,’ and you’ll be able to build your merits as a researcher.” [Interview participant; Sweden] - Interestingly, the United States spoke about offering validation data sets as a separately commercialized entity. This challenged the assumption that AI companies would want to be involved at all stages of the initiative and suggests a feasible model whereby different data offerings give value to different users according to their needs. |
|  | M. Future-proofing mechanisms for data sharing | - Some countries had put in place mechanisms to future-proof the use of the data they collected and used as part of their initiative, both to rescind data access and extend it to future offerings. For example, the United States has a master agreement that enables them to quickly include additional data from sites without further negotiation and creation of new data-sharing arrangements. Sweden has also built-in contractual mechanisms to revoke data sharing in the future if necessary. |
| **Incentives** | | |
|  | N. Focus on public and social good | - Most countries shied away from narratives or activities associated with commercialization, preferring to focus on the public and social good of the work they were doing. The framing of how participants were incentivized to take part focused on the value of data sharing to reap benefits from access to a larger data set and a cultural mandate for big data to be as accessible as possible:   - “In our country our government decided if the data is created using the national budget then our data should be shared among the researchers as much as possible.” [Interview participant; Japan] - None of the countries have considered the environmental sustainability or impacts of the initiatives. |
| **Building trust** | | |
|  | O. Transparency and communication | - All countries recognized the importance of being able to explain why data are shared, how data are used, and what is the benefit to the end user and health system. This links to the importance of having a clear purpose and narrative with demonstrable benefit. - Initiatives have a responsibility of clear communication to avoid AI being an esoteric domain and disregarding the public’s potential interest and ability to understand. Although some countries were dismissive of public interest (Sweden), others (Japan and Canada) were active in engaging and understanding that the initiatives needed to account for public perception, including public presentations of their work. In addition, Hong Kong included public impact as an assessment criterion within their initiative when selecting projects and teams that can access the data. |
|  | P. Keeping humans in the loop | - All countries, including the NHS AI Lab discovery team during the workshop, maintained that humans were “in the loop” when it came to data sharing and the development of AI models. “Human-in-the-loop” refers to maintaining human oversight in the development and implementation of algorithms, whereby a human can intervene and determine the outcome of a process or event. However, there was an undertone that, eventually, as AI development was normalized or became mainstream and accepted, they could move away from having humans in the loop in some cases. |
| **Law, policy, and regulation** | | |
|  | Q. Advocating for policy, regulatory, and legal frameworks that are fit for purpose | - All countries recognized that, to varying extents, current policies and regulations are not fit for purpose for AI development and implementation. Many countries described the requirement to work within existing legal and regulatory constraints that had shaped the platform structure and function (such as Canada’s federated platform). Japan stressed that, because of existing laws that were in the process of changing, they are unsure of how to extend their platform for use in the private sector. - Hong Kong highlighted that, as all the basic regulatory and policy structures were in place, they were able to focus on making data accessible and adding tools for researchers to interact with the data. Hong Kong shared that having a supportive and fit-for-purpose regulatory and policy environment gave them a lot of freedom to focus on the initiative. - There was a difference in mindset between countries that spoke solely about operating within existing constraints and those that were insistent on working as creatively as possible within these bounds while also expressing a desire to change them. Although Canada doubled down on creating technology that aligned with the existing policy and regulatory requirements, Sweden looked to skillfully navigate and expand what people could do within the regulations and how to drive change. An example the interview participant shared was summarizing policies for users and sharing practical steps for meeting the policy requirements, such as anonymizing images. |

^a^AI: artificial intelligence.

^b^DHDP: Digital Health and Discovery Platform.

^c^AIDA: Analytic Imaging Diagnostics Arena.

^d^NHS: National Health Service.

^e^IP: intellectual property.

^f^DOI: digital object identifier.
